# Unveiling the Link: A Comprehensive Narrative Review of the Relationship Between Type 1 Diabetes Mellitus and Celiac Disease

**DOI:** 10.7759/cureus.47726

**Published:** 2023-10-26

**Authors:** Sanvi Arora, Ayush Tayade, Tanya Bhardwaj, Swanand S Pathak

**Affiliations:** 1 Medicine, Jawaharlal Nehru Medical College, Datta Meghe Institute of Higher Education and Research, Wardha, IND; 2 Pharmacology, Jawaharlal Nehru Medical College, Datta Meghe Institute of Higher Education and Research, Wardha, IND

**Keywords:** autoimmune disease, genetic risk factors, celiac disease and diabetes, celiac disease and autoimmunity, type 1 diabetes mellitus (t1dm)

## Abstract

Type 1 diabetes mellitus (T1DM) is an autoimmune condition with a genetic predisposition. It has underlying autoimmune destruction of the pancreatic cells that produce insulin. It is often accompanied by other autoimmune conditions. This article focuses on celiac disease (CD), also an autoimmune disease. It is caused by gluten exposure. Both these conditions have genetic predisposing factors. Apart from the genetic background, aberrant small intestine immune response, inflammation, and different grades of enteropathy present in T1DM and CD are the same. With a mean frequency of 8%, the CD frequency of T1DM ranges from 3 to 16%. All T1DM patients should undergo serological testing for CD using antibodies to tissue transglutaminase at the time of T1DM onset. Individuals with T1DM and those accompanied by CD must follow a diet with no gluten. To outline the steps that can avert the development of these disorders and reduce the morbidity of the affected people, a complete understanding of the intricate pathophysiology of T1DM and its connection to CD has been undertaken in this review. The use of resources, such as PubMed and Google Scholar, has made this possible.

## Introduction and background

One of the most prevalent chronic metabolic disorders in the young population is diabetes. Type 1 diabetes mellitus (T1DM) is characterized by a lack of insulin since the beta cells that produce insulin in the pancreas have been destroyed. T1DM is detected more frequently in people who have a poor genetic propensity. The primary gene for susceptibility is found on chromosome 6's human leukocyte antigen (HLA) region, and it has a high association with alleles DR3, DR4, DQA1-0501, DQA1-0501, DQA1-0301, and DQB1-0302. The HLA complex is thought to be responsible for 40-50% of T1DM risk [1].

Since diabetes is an autoimmune condition, it is seen to impact other organs, which can result in making the management more difficult to overcome [2]. T1DM is accompanied by several autoimmune disorders: Hashimoto's thyroiditis, Graves' disease, Addison’s disease, celiac disease (CD), and autoimmune gastritis [3-7].

An autoimmune reaction directed against pancreatic beta cells characterizes T1DM, also an autoimmune condition. Anti-islet autoantibodies arise before the clinical need emerges, and T1DM is usually accompanied by other autoimmune diseases [2]. This article focuses on the association between T1DM and CD, its pathophysiology, association, serodiagnosis, and management. The review aims to thoroughly and critically assemble all available data supporting the link between the two autoimmune conditions.

## Review

Methodology

For gathering all the required papers for this review article, a thorough literature search technique was needed in the methodology section of this narrative review. Google Scholar and PubMed were the two electronic databases accessed. The following keywords were used: "genetic risk factors," "type 1 diabetes mellitus," "autoimmune disease," "celiac disease and diabetes," and "celiac disease and autoimmunity." All the authors unanimously carried out a thorough search.

**Figure 1:**
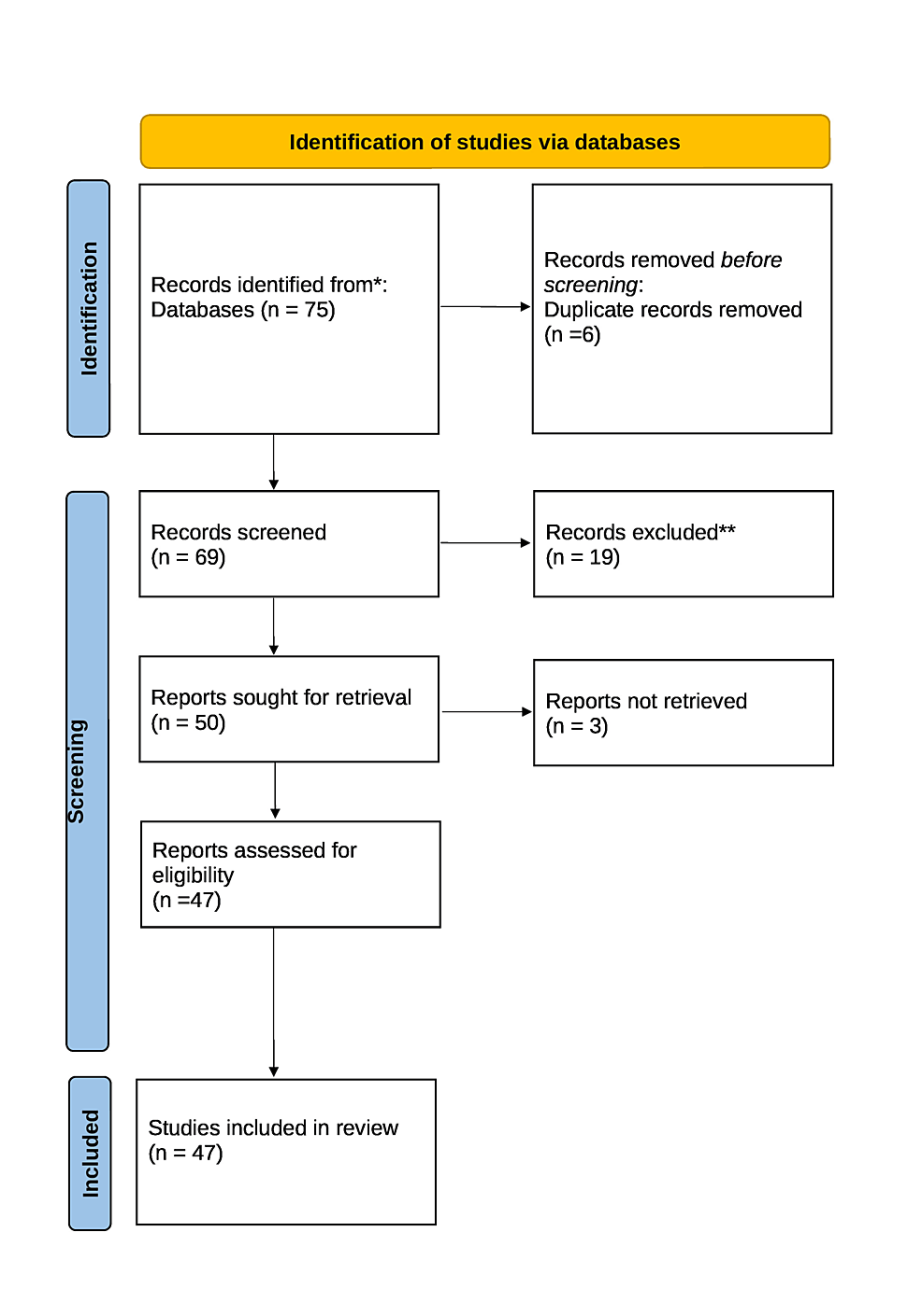
PRISMA methodology Adopted from Preferred Reporting Items for Systematic Reviews and Meta-Analyses (PRISMA)

The inclusion of both original research articles and review papers was taken into consideration. To ensure that the inclusion of studies satisfies the established criteria, the selection procedure comprised of evaluating article titles, abstracts, and full texts. Conflicts over the choice of studies were settled by author consensus. The method for choosing the articles we utilized in our study is shown in Figure 1.

What is T1DM?

Insulin shortage results from the autoimmune disease T1DM, caused by the death of the pancreatic beta cells producing insulin [8]. Insulin is a significant anabolic hormone that affects growth and impacts glucose, lipids, proteins, and minerals metabolisms. Thus, T1DM manifests as a systemic illness with the phenotype of hyperglycemia.

A series of research have been conducted tracing the factors affecting the onset of T1DM. Genetic factors play a significant role. The primary gene for susceptibility is found on chromosome 6's HLA region, and it has a high association with alleles DR3, DR4, DQA1-0501, DQA1-0201, DQA1-0301, and DQB1-0302. The HLA complex is thought to be responsible for 40-50% of T1DM risk [2]. HLA-linked T1DM causes increasing insulin insufficiency and hyperglycemia. Type 1A (immune-mediated) and type 1B (idiopathic) T1DM were the categories used by the American Diabetes Association Expert Committee in 1997 [9].

Atypical T-cell activation causes insulitis and the development of antibodies against cells, which may serve as helpful indicators of immunological destruction. Type 1A diabetes is caused by cell-mediated autoimmune killing of cells in the pancreas. The islet cell antigens insulin (IAA), insulinoma antigen 2 (IA-2), glutamic acid decarboxylase 65 kDa isoform (GAD-65), and zinc transporter 8 (ZnT8) are targets of the majority of the autoantibodies seen in people with type 1A diabetes [10].

Without pancreatic cell antibodies, type 1B diabetes is a heterogeneous condition with an uncertain cause of negative cell apoptosis. Japanese researchers have described a fulminant variant of type 1B diabetes having accompanying exocrine pancreatic dysfunction, presumably caused by a viral infection [11].

Pathophysiology of diabetes 

Research on the natural course of T1D reveals that islet-cell autoimmunity triggered by T-cells is more common than previously thought. T1D's pathogenesis is more complex than initially thought. It has been observed that various factors are at play, based on evidence from studies from the past 20 years. To name a few, they are genetic, epigenetic, and external factors [12]. In people with a genetic predisposition, environmental circumstances are what will cause the condition [13].

In actuality, the pancreatic islet-cell vulnerability results in negative cell stress. There is the production of tumor cell-generated self-antigen, called neoantigen. These neoantigens are affected by the immune system. Therefore, a confluence of different immune molecular events frequently causes localized inflammation, also known as insulitis, which culminates in a noticeable and persistent loss of cell functions. The pancreas can avoid the autoimmune attack initially because very few autoantigens have been identified.

In addition, the death of the islets is held down by processes, such as changes to the surface antigens [14,15]. A decline in cell volume and the malfunctioning of the cells contribute to the start of hyperglycemia [16]. Recent research describes the role of cell-associated immunogenicity in addition to the autoimmune component. This role could be linked to genetic or environmental variables [16].

HLA connections exist for T1DM. The HLA on chromosome 6 was the first locus identified by candidate gene research as being related to the disease, and it is accountable for around half of the hereditary basis of T1DM. A large proportion (90%) of children with T1DM have the HLA gene combinations DR4-DQ8 and DR3-DQ2, which are of particular significance [17]. Just under 1% of youngsters with T1DM carry the protective third haplotype, DR15-DQ6, although over 20% of the general population do. The genotype (DR4-DQ8/DR3-DQ2) that combines the two susceptibility haplotypes (DR4-DQ8/DR3-DQ2) is most prevalent in young children, where the disease manifests itself relatively early in life [18]. The second majorly significant genetic susceptibility factor for T1DM is the insulin gene on chromosome 11, which results in 10% of genetic susceptibility [19]. There are a minimum of 15 more loci linked to T1DM, according to whole genome screening results over the past 10 years [10].

Diabetes and autoimmune system disorders

There is proof that having T1DM raises the risk of developing other autoimmune conditions. It is connected to these disorders' genetic predisposition to growth. The autoimmune process that is growing in pancreatic beta cells can also impact other organs, which leads to the emergence of autoimmune disorders that are specific to those organs, or it can damage a variety of non-specific tissues and organs, resulting in autoimmune diseases that are not particular to those organs [20]. Anti-islet autoantibodies arise before the clinical disease emerges, and T1DM is usually accompanied by some autoimmune diseases [2].

T1DM is most commonly linked to autoimmune thyroid disorders (Hashimoto's thyroiditis and Graves' disease), Addison's disease, CD, and autoimmune gastritis, but not to rheumatoid arthritis or systemic lupus erythematosus [2,4,5]. Other autoimmune disorders that are silent and asymptomatic in T1DM patients can be found with serological screening [9]. The above information has been tabulated in Table 1. This association makes the management of T1DM difficult.

**Table 1:** Prevalence of autoimmune disorders associated with T1DM Source: [2,4,5] T1DM: Type 1 diabetes mellitus

Autoimmune disorders associated with T1DM	Prevalence
Hashimoto's thyroiditis and Graves' disease	17–30%
Addison's disease	0.2%
Celiac disease	8%
Autoimmune gastritis	5–10%
Rheumatoid arthritis	1.2%
Systemic lupus erythematosus	1.15%

Celiac disease

Gluten, a protein in wheat, rye, barley, and other grains, can cause CD, an inflammatory condition that manifests as a multisystem inflammation with enteropathy as its hallmark [8]. Antireticulin (ARA), anti-endomysial (EmA), antigliadin (AGA), and anti-tissue transglutaminase (ATG) autoantibodies are explicitly produced in response to gluten, and intestinal villi also shrink.

Silent, possible, latent, and classical illness subtypes have all been identified. The incidence of underlying HLA haplotypes among the people and per capita wheat consumption are the critical factors of occurrence, which vary with gender, age, and geographic region. A minor gender bias to the benefit of women exists [21].

One of the most prevalent autoimmune diseases among people with T1DM is CD. With an average incidence of 8% and a range from 3% to 16%, CD is much more seen in people with T1DM than in the overall population [2,5]. The diagnosis of T1DM is made before it is associated with CD in more than 90% of cases [22]. In addition, when set alongside the general population, juvenile individuals with T1DM are more likely to have CD diagnosed [23].

Age and sex are also risk factors for this illness. It is well known that it affects ladies and children more frequently than males and adults [24]. Although environmental factors have a significant role in causing CD, CD's high heritability and robust HLA connection stand out [25].

In CD, HLA molecules with gluten compounds that bind to it activate T cells, one of the most crucial functions of CD4+ T cells [26]. Ninety percent (90%) of Caucasian CD patients have the HLA-DQ2.5 haplotype (encoded by the DQA1*05:01 and DQB1*02:01 alleles) either in cis or trans positions. The remainder holds either HLA-DQ8 (encoded by the DQA1*03:01 and DQB1*03:02 alleles), HLA-DQ2.2 alone (encoded by the DQB1*02:02 allele), or HLA-DQ7 alone (encoded by the DQA1*05:01 allele). A “gene dose effect” exists, which is known to impact clinical phenotypes and individual behavior to a T cell targeted therapy. This effect is connected to the number of copies of the DQB1*02 allele. This impact is thought to be caused by the fact that gluten delivered by APCs in HLA-DQ2.5 homozygous (dual versions of DQB1*02) person can trigger a T-cell response that is at least four times greater than in HLA-DQ2.5 heterozygous (one copy of DQB1*02) persons [27].

Association between the autoimmune diseases: T1DM and CD

As was already noted, HLA is associated with T1DM. Two HLA gene (or haplotype) combinations are particularly significant: 90% of kids with T1DM had the mutations DR4-DQ8 and DR3-DQ2 [17]. HLA-, HLA-DQ2-, or DQ8-positive people usually have CD. The linking factor between T1DM and CD has existed since 1969 [28].

HLA genes and non-HLA genes are predisposing factors linked to both disorders. Approximately 50% of the possibility of T1DM is caused by genetic factors [29]. HLA-DQ2 and DQ8 have the most evident correlation with T1DM-related genes. The increased expression of the HLA-DQ2 and HLA-DQ8 antigens makes people more likely to develop both disorders [30]. The risk of CD has been associated with specific HLA alleles. Therefore, CD is more likely to manifest in T1DM patients [29,31].

A similar genetic association is seen in the association of T1DM, Hashimoto's thyroiditis, and Grave's disease. The predisposing factor shared between the autoimmune thyroid diseases and T1DM is HLA antigens DQ2 (DQA1∗0501-DQB1∗0201) and DQ8 (DQA1∗0301-DQB1∗0302). Compared with the association between T1DM and CD, there is a presence of antithyroperoxidase and antithyroglobulin antibodies, which is not seen in the former. Thus, the treatment varies when T1DM coexists with Hashimoto's thyroiditis and Grave's disease, which involve levothyroxine monotherapy and corticosteroids [2].

Gluten and T1DM are linked, according to studies in animals. It has been hypothesized that gluten impacts negative cells and may be connected to the emergence of T1DM. Other factors, including adaptive and innate immune systems, pro-inflammation, intestinal microbiota, and permeability, may have an additional impact [31-36].

The pancreas is affected by a protein called giladin [13], potentially causing inflammation and cell stress [9,10]. Giladin is present in wheat and other gluten-containing grains. Applying a diet without gluten for life decreased the prevalence of T1DM in mice from 64% to 15% [12,14].

Besides the genetic link between T1DM and CD, evidence indicates that consuming more gluten may make T1DM more common. For instance, research showed that pregnant women who ingested more gluten during their pregnancy had a correspondingly higher T1DM risk in their offspring [37]. The chance of developing diabetes increases when gluten is added to a baby's diet either after seven months or before the baby is four months old. According to Norris et al.'s (2003) theory, CD promotes diabetes through increasing gut permeability caused by gluten-mediated inflammation [38,39]. According to specific data, a gluten-free diet (GFD) may have a favorable impact on the pathophysiology, onset, and clinical progress of T1DM [40]. Human studies are uncommon in any case.

The occurrence of CD or T1DM has been linked to several environmental variables. T1DM progression has been associated with enterovirus infections, notably Coxsackie [41,42]. Rotavirus has been linked to a higher risk of CD and T1DM [43]. Furthermore, both disorders appear to be influenced by changes in intestinal permeability and microbiota [44].

Management and diagnosis

Patients with T1DM and CD may present with nonclassical or asymptomatic diseases. Therefore, there is a requirement for periodic screening of T1DM patients for CD. Serological autoimmune markers, like the IgA anti-tissue transglutaminase (TTG) and IgA anti-endomysial (EMA) antibodies, are currently employed for routine CD screening to recognize different kinds of CD since they are susceptible and particular [23]. Throughout the condition, diabetic people must have multiple serological screenings for CD. The silent or prospective phase of CD, which occurs before the development of clinical manifestations, is when it is most frequently discovered in children with T1DM [45,46].

In people with diabetes with CD, eliminating gluten from the diet restores the structural makeup of the small intestine mucosa, enabling normal nutritional absorption and alleviating symptoms without significantly slowing the progression of diabetes [5].

Compared to people with diabetes without CD, diabetic individuals with untreated CD had a lower body mass index and reduced HbA1c levels. Untreated CD can lead to weight loss, which can, in turn, help with glycemic management. However, following a GFD increases the amount of nutrients absorbed at the intestinal threshold, increasing the insulin requirement [2]. A diet free of gluten has been demonstrated to reduce the frequency of hypoglycemia episodes while maintaining an appropriate amount of glycosylated hemoglobin [5].

The development of the metabolic syndrome can be facilitated by the enhancement of intestinal absorption and frequent ingestion of commercial foods having no gluten, specifically those high in carbohydrates and lipids, which raises the likelihood of heart-related illnesses even more than the presence of T1DM alone. Contrarily, adhering to such a diet may help prevent the long-term effects of CDs, such as malnutrition, osteoporosis, intestinal lymphoma, and intestinal adenocarcinoma. Exogenous insulin supplementation and a GFD are the way to go for the treatment of coexisting conditions [47].

## Conclusions

The article summarizes that T1DM affects children most frequently. T1DM is often associated with other autoimmune disorders, namely, Grave's disease and Hashimoto's thyroiditis, autoimmune gastritis, and CD. T1DM and CD have an underlying genetic risk factor that links the two and are often seen together in children and adolescents. When T1DM and CD coexist, glucose metabolism is compromised, effective insulin therapy is hampered, and diabetes management is worsened. Serodiagnosis is done for diagnosing purposes. Patients are advised to a GFD. Individuals with autoimmune illnesses must closely monitor their immune system reactions since they may impact other organs.
